# Molecular characteristics of four Japanese cases with *KCNV2* retinopathy: Report of novel disease-causing variants

**Published:** 2013-07-20

**Authors:** Kaoru Fujinami, Kazushige Tsunoda, Natsuko Nakamura, Yu Kato, Toru Noda, Kei Shinoda, Kaoru Tomita, Tetsuhisa Hatase, Tomoaki Usui, Masakazu Akahori, Takeshi Itabashi, Takeshi Iwata, Yoko Ozawa, Kazuo Tsubota, Yozo Miyake

**Affiliations:** 1 National Institute of Sensory Organs, National Tokyo Medical Center, Tokyo, Japan; 2 Department of Ophthalmology, Keio University, School of Medicine, Tokyo, Japan; 3 School of Medicine, Teikyo University, Tokyo, Japan; 4 Heiwa Ganka Clinic, Tokyo, Japan; 5 Graduate School of Medical and Dental Sciences, Niigata University, Niigata, Japan; 6 Akiba Eye Clinic, Niigata, Japan; 7 Aichi Medical University, Aichi, Japan

## Abstract

**Purpose:**

To describe the molecular characteristics of four Japanese patients with cone dystrophy with supernormal rod responses (CDSRR).

**Methods:**

Four individuals with a clinical and electrophysiological diagnosis of CDSRR were ascertained. The pathognomonic findings of the full-field electroretinograms (ERGs) included a decrease in the rod responses, a square-shaped a-wave, an excessive increase in the b-wave in the bright flash responses, and decreased cone-derived responses. Mutational screening of the coding regions and flanking intronic sequences of the potassium channel, subfamily V, member 2 (*KCNV2*) gene was performed with bidirectional sequencing. The segregation of each allele was confirmed by screening other family members. Subsequent in silico analyses of the mutational consequences for protein function were performed.

**Results:**

There were two siblings from one family and one case in each of the two families. One family had a consanguineous marriage. Mutational screening revealed compound heterozygosity for the two alleles, p.C177R and p.G461R, in three patients, and homozygosity for complex alleles, p.R27H and p.R206P, in one patient from the consanguineous family. There were three putative novel variants, p.R27H, p.C177R, and p.R206P. The four variants in the families with *KCNV2* were highly conserved in other species. In silico analyses predicted that all of the missense variants would alter protein function.

**Conclusions:**

Biallelic disease-causing variants were identified in four Japanese patients with CDSRR suggesting that the pathognomonic electrophysiological features are helpful in making a molecular diagnosis of *KCNV2*. Three novel variants were identified, and we conclude that there may be a distinct spectrum of *KCNV2* alleles in the Japanese population.

## Introduction

Patients with cone dystrophy and supernormal rod electroretinograms (ERGs) were first reported in 1983, and the abnormality in the ERGs indicated a progressive degeneration of the cone photoreceptors associated with unique rod system abnormalities [1]. More detailed characteristics of this rare, autosomal recessive condition were reported in later studies, and the disease was named cone dystrophy with supernormal rod responses (CDSRR; MIM #610356) [2-8].

Most cases with CDSRR typically present in the first two decades of life with reduced visual acuity, abnormal color vision, and photophobia [8-11]. Night blindness is a later feature of the disorder [8]. The fundus appearance is variable, with some having a normal peripheral retina and a range of macular abnormalities [8-10]. The pattern of the autofluorescence (AF) images is also variable: Young cases have either a normal pattern or small parafoveal ring enhancements, while older cases have a narrow high-signal annulus that can encircle a central atrophic area of the retinal pigment epithelium (RPE) [6,12]. Recently, spectral domain optical coherence tomography (SD-OCT) and adaptive optics scanning laser ophthalmoscope (AOSLO) studies have described morphological changes of the fovea even at the early stages [10,13,14].

The electrophysiological findings are pathognomonic of CDSRR, and they assist in its early diagnosis [3,5,8-12,15-17]. The light-adapted ERGs are usually delayed and decreased in keeping with a generalized cone system dysfunction. There is also a unique rod system abnormality; the dark-adapted ERGs elicited by dim flashes are markedly decreased and delayed, and increasing the flash intensity results in an excessive increase in the b-wave amplitude accompanied by a shortening of the peak time of the b-wave [8,9,11]. A square-shaped a-wave trough of the dark-adapted bright flash ERGs is also a characteristic feature of this disorder [9,11].

CDSRR has been shown to be caused by mutations in the potassium channel, subfamily V, member 2 (*KCNV2*) gene (MIM# 607,604), which encodes a voltage-gated potassium channel subunit, Kv8.2 [18]. This silent subunit is expressed in rod and cone photoreceptors [18-20], and is thought to assemble with other K^+^ channel subunits such as KCNB1, KCNC1, and KCNF1. These subunits form functional heteromeric channels with altered properties that have a narrowed membrane potential for activation and slow inactivation kinetics [19]. Eventually, these kinetic properties result in transient hyperpolarization overshoots on rapid changes in the inward currents [19]. A deficiency of Kv8.2 by a mutation in *KCNV2* may affect the characteristics of the I_kx_ as first described in amphibian photoreceptors [21]. This deficiency may influence the photoreceptor membrane potential. However, the underlying mechanisms that fully explain the clinical features of CDSRR are still not determined.

More than 50 different disease-causing variants in *KCNV2* have been reported: small insertion and deletion changes or large deletions that constitute a protein truncation and single nucleotide changes with amino acid substitutions [9,10,13,14,16,18,22,23]. Three small case series describe the clinical features of CDSRR in East Asians [3,5,15]; however, molecular genetic studies of these populations have not been published. Thus, the purpose of this study was to determine the molecular genetic characteristics from the clinical and electrophysiological findings of four Japanese patients who were diagnosed with CDSRR.

## Methods

### Subjects

Four subjects who were diagnosed with CDSRR from the clinical and electrophysiological findings were ascertained at the National Institute of Sensory Organs, National Tokyo Medical Center, Tokyo, Japan and Niigata University, Niigata, Japan. The natural history of these four patients has been partially reported recently [24]. The procedures used were approved by the ethics committee of each institution, and all procedures were performed in accordance with the principles of the Declaration of Helsinki. Informed consent was obtained from all experimental subjects for all procedures.

### Clinical assessment

A complete medical history was obtained, and a comprehensive ophthalmological examination was performed on all patients. The photophobia and night blindness episode was obtained on direct questioning. The clinical assessments included measurements of the best-corrected visual acuity (BCVA), dilated ophthalmoscopy, color fundus photography, AF imaging, OCT, and electrophysiological recordings. AF images were obtained with the HRA 2 confocal scanning laser ophthalmoscope (Heidelberg Engineering, Heidelberg, Germany; excitation light, 488 nm; barrier filter, 500 nm; field of view, 30×30°) [25]. The OCT images were obtained with SD-OCT (Cirrus HD-OCT, versions 4.5 and 5.1; Carl Zeiss Meditec, Dublin, CA) [26].

### Electrophysiological assessments

Full-field ERGs were recorded from the four patients with the minimum standard protocol of the International Society for Clinical Electrophysiology of Vision (ISCEV) [27]. The ERG examination included the following: (i) dark adapted dim flash 0.01 cd•s•m^−2^ (DA 0.01), (ii) dark adapted bright flash 30.0 cd•s•m^−2^ (DA 30.0), (iii) light adapted 3.0 cd•s•m^−2^ at 2 Hz (LA 3.0), and (iv) light adapted 3.0 cd•s•m^−2^ 30 Hz flicker ERG (LA 3.0 30Hz). The extended protocol included the recording of the dark-adapted ERGs elicited by stimulus intensities of 0.001 cd•s•m^−2^, 0.01 cd•s•m^−2^, 0.3 cd•s•m^−2^, 3.0 cd•s•m^−2^, and 30.0 cd•s•m^−2^. Two of the four patients were also recorded with the extended protocol. An excessive or disproportionate increase in the dark adapted b-wave with increasing flash intensity was assessed in these two patients, according to the previous report [9].

### Mutational screening

After informed consent was obtained, blood samples were collected in EDTA tubes from each subject, and the DNA was extracted with a DNA extraction kit (QIAamp DNA Blood Maxi Kit; Qiagen, Venlo, the Netherlands). All exons and exon–intron boundaries were amplified with polymerase chain reaction (PCR), and the primer sequences used are shown in Table 1. PCR was performed with 20 μl volume containing 0.5 Unit Taq polymerase (PrimeStar GXL DNA polymerase, Takara, Tokyo, Japan). The sequence was determined based on the dideoxy terminator method using an ABI PRISM 3100×l Genetic Analyzer (Applied Biosystems, Foster City, CA) according to the manufacturer’s protocol. The SeqScape Software version 2.5 (Applied Biosystems) was used to analyze the sequence alignment. Bidirectional Sanger sequencing was also performed in other family members of the proband, to confirm the segregation of the alleles.

**Table 1:** Primer Sequences and Conditions for *KCNV2* Mutational Screening.

Primer	Sequence (5'–3')	Product size (bp)	PCR annealing (°C)
E1aF	AGGACCTGAGAAGGGGCAGCT	831	71
E1aR	TCCAGGAGGCGGAGGAACTCT		
E1bF	CCCTGCTGTCCACGCTGAATG	799	71
E1bR	CAGCGTGGGTAAGGTGGGTCA		
E1cF	AAGATCCAGCACGAGCTGCGC	841	65
E1cR	ATGGATGTCAAAAGTGGTGGA		
E2aF	AGCTTCTGTTCTTTTCATGAC	624	63
E2aR	GTCTCATAGTTGCTCTGTGTT		

bp = base pairs.

### Molecular genetic analyses

All of the missense variants identified were analyzed using two software prediction programs, Sorting Intolerant from Tolerance (SIFT) and PolyPhen2 [28,29]. The predicted effects on splicing of all missense variants were assessed with the Human Splicing finder program version 2.4.1. The allele frequency of each variant was estimated with the Exome Variant Server (NHLBI Exome Sequencing Project, Seattle, WA). A multiple sequence alignment program for DNA or proteins, the Clustal Omega, was applied to confirm an evolutionary conservation. Likely non-disease-causing variants (polymorphisms) were also analyzed with the same protocol applied to likely disease-causing variants.

## Results

**Figure 1:**
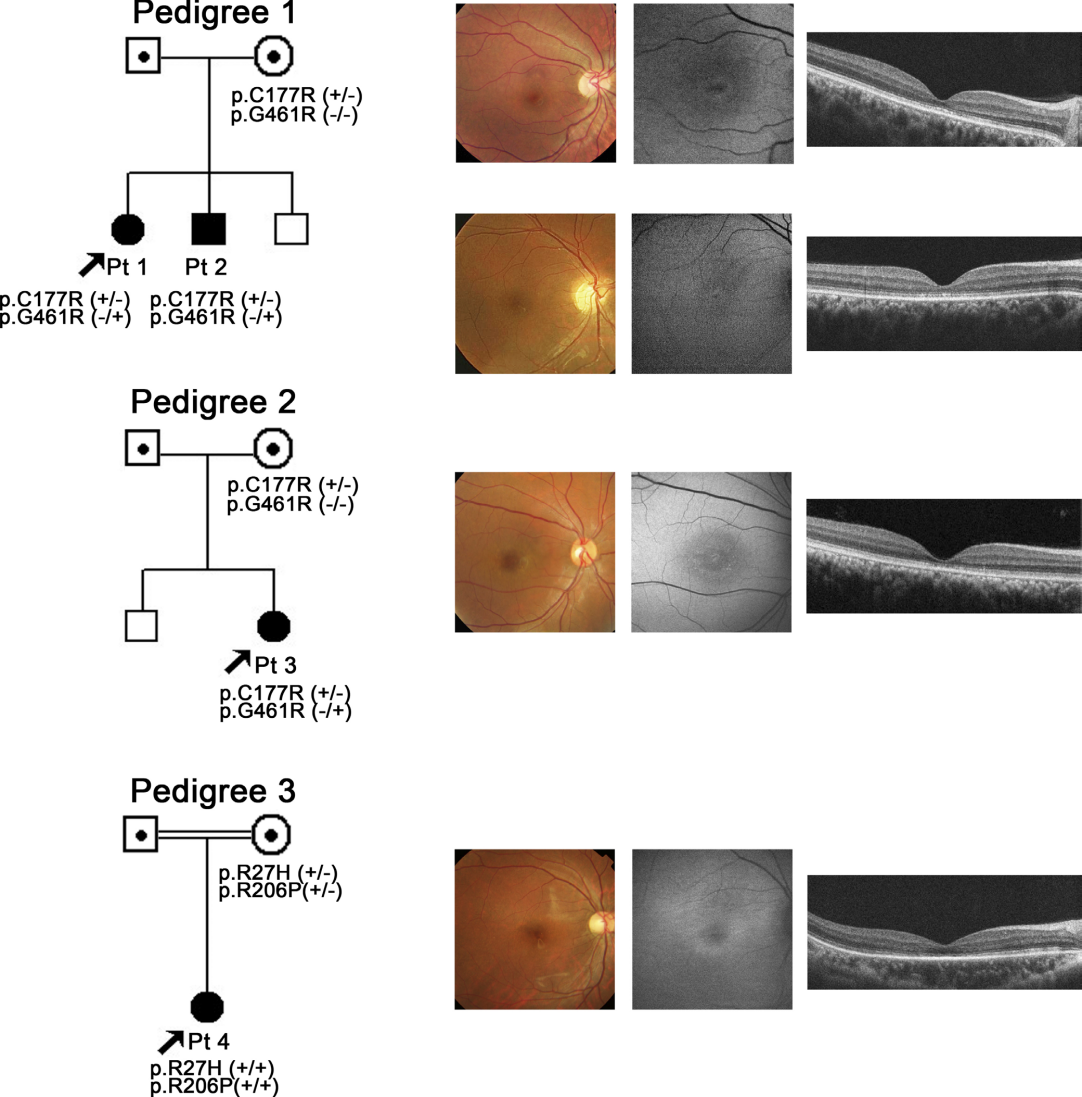
Pedigree and retinal imaging of each patient with potassium channel, subfamily V, member 2 retinopathy. Pedigrees with molecular status of the three families with potassium channel, subfamily V, member 2 (*KCNV2*) retinopathy are shown on the left. Retinal images including color fundus photographs, autofluorescence images, and spectral domain optical coherence tomography are presented on the right. Images of patient 1 (top row), patient 2 (second row from top), patient 3 (third row from top), and patient 4 (bottom row) are shown.

The demographic features of the four individuals from three families with CDSRR are summarized in Table 2. There were two siblings (patients 1 and 2) in one family and one case in each of the two families (patients 3 and 4). The pedigree of each family is shown in Figure 1, and a consanguineous marriage was present in family 3.

**Table 2:** Summary of demographics, clinical findings and molecular status for four Japanese patients with *KCNV2*-retinopathy

Pt, FM, gender	Onset of disease, age at examination (years)	VA		Fundus		AF		OCT		Mutation status
		RE	LE	RPE mottling	Subtle patchy granular flecks	Ring enhancement	Patchy granular foci of high signal	Absence of COST	Deficit of IS/OS	
1, 1, F	9, 23	0.7	0.8	Macula	ND	Fovea	ND	Fovea	ND	Compund heterozygous [c.529 T>C, p.Cys177Arg]; [c.1381G>A, p.Gly461Arg]
2, 1, M	5,17	0.7	0.7	Macula	ND	Fovea	ND	Fovea	ND	Compund heterozygous [c.529 T>C, p.Cys177Arg]; [c.1381G>A, p.Gly461Arg]
3, 2, F	3,21	0.1	0.1	Macula	Macula	Fovea	Macuala	Macula	Fovea	Compund heterozygous [c.529 T>C, p.Cys177Arg]; [c.1381G>A, p.Gly461Arg]
4, 3, F	2, 17	0.1	0.08	Macula	ND	Para-fovea	ND	Macula	Fovea	Complex homozygous [c.80 G>A, p.Arg27His]; [c.617 G>C, p.Arg206Pro]

Pt = Patient; FM = family number; VA = logMAR visual acuity; RE = right eye; LE = left eye; RPE = retinal pigment epithelium; AF = autofluorescence; COST = cone outer segment tip line; IS/OS = photoreceptor inner and outer segment junction; ND = not detected. The affected area of each finding is based on the color fundus photographs, AF images, and the OCT images in each column.

### Clinical findings

The age of the patient at the time of the examination was 23, 17, 21, and 17 years with the age of disease onset at 9, 5, 3, and 2 years (Table 2). Three patients complained of photophobia (patients 1, 2, and 4), and all four patients had night blindness. Patient 4 had had mild nystagmus since age 2 years. The decimal BCVA of the four patients ranged from 0.08 to 0.8, and the BCVA of patients 1 and 2 was better than 0.7 in each eye.

The findings obtained from the color fundus photographs, AF images, and SD-OCT images are summarized in Figure 1 and Table 2. The fundus photographs showed mottling of the RPE at the macula in all four patients with subtle patchy granular flecks at the macula in patient 3. A ring enhancement of the AF signal was detected in the AF images of all four patients; three subjects had it centered on the fovea (patients 1, 2, and 3), and one had it at the parafovea (patient 4). In patient 3, the ring enhancement at the fovea was surrounded by patchy granular foci of the high AF signal at the macula.

SD-OCT demonstrated abnormalities in the outer retinal layers in all four patients. The cone outer segment tip line was absent in the macular area in all patients. The photoreceptor inner and outer segment junction line was discontinuous at the fovea in patients 3 and 4, and thinning of the outer retina was detected at the fovea in all four patients.

### Electrophysiological assessments

**Figure 2:**
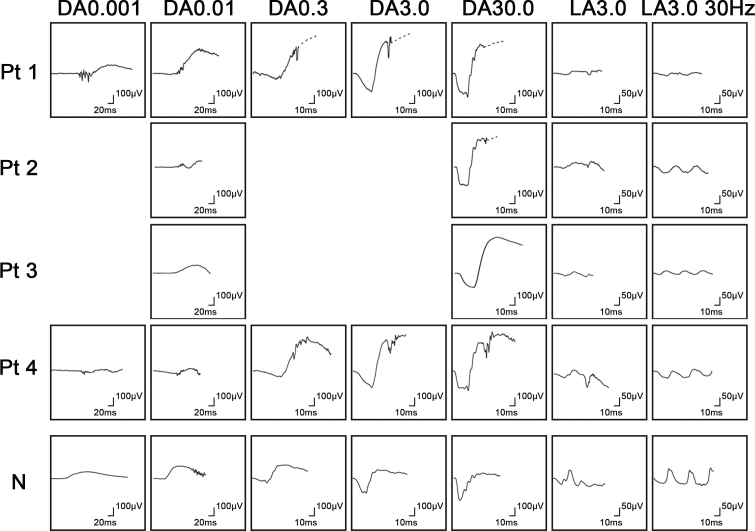
Electrophysiological findings of each patient with potassium channel, subfamily V, member 2 (*KCNV2*) retinopathy. Full-field electroretinograms (ERGs) of patient 1 (top row), patient 2 (second row), patient 3 (third row), and patient 4 (forth row) are shown. The ERGs from a normal control (bottom row) are also shown for comparison. All four patients underwent full-field ERG testing with the minimum standards of the International Society for Clinical Electrophysiology of Vision (ISCEV): (i) dark adapted dim flash 0.01 cd•s•m^−2^ (DA 0.01), (ii) dark adapted bright flash 30.0 cd•s•m^−2^ (DA 30.0), (iii) light adapted 3.0 cd•s•m^−2^ at 2 Hz (LA 3.0), and (iv) light adapted 3.0 cd•s•m^−2^ 30 Hz flicker ERG (LA 3.0 30Hz). The extended protocol was applied to two subjects (patients 1 and 4), including the recording of dark-adapted ERGs to an intensity series of flashes; 0.001 cd•s•m-^2^, 0.01 cd•s•m^−2^, 0.3 cd•s•m^−2^, 3.0 cd•s•m^−2^, and 30.0 cd•s•m^−2^.

The electrophysiological findings are summarized in Table 3, and the ERGs are shown in Figure 2. The full-field ERGs were recorded with the minimum ISCEV standard from patients 2 and 3, and extended protocol full-field ERGs including the dark-adapted ERGs elicited by an intensity series were obtained from patients 1 and 4.

**Table 3:** Electrophysiological Findings of Four Japanese Patients with KCNV2-retionpathy

Pt	DA 0.01		DA 30.0				Square shaped a-wave	Excessive enlargement of b-wave in the extended protocol	LA 3.0			LA 3.0 30Hz		
	Amp (μv)	PT (ms)	A-wave		B-wave				A-wave	B-wave		B-wave		
			Amp	PT	Amp	PT			Amp	PT	Amp	PT	Amp	PT
1	N	Del	N	Del	Super N	NA	(+)	(+)	Sub N	Del	Sub N	UD	UD	UD
2	UD	UD	N	Del	Super N	NA	(+)	NA	Sub N	Del	Sub N	Del	Sub N	Del
3	Sub N	Del	N	Del	Super N	N	(+)	NA	Sub N	Del	Sub N	Del	Sub N	N
4	Sub N	Del	N	Del	Super N	NA	(+)	(+)	Sub N	Del	Sub N	Del	Sub N	Del

Pt = patient; Amp = amplitude; PT = peak time; N = normal; UD = undetectable response; Sub N = subnormal; Del = delayed response; Super N=supernormal response; NA = not available. Full-field electroretinography (ERG) incorporating the standards of the International Society for Clinical Electrophysiology of Vision (ISCEV) included: (i) dark adapted dim flash 0.01 cd•s•m^-2^ (DA0.01), (ii) dark adapted bright flash 30.0 cd•s•m^-2^ (DA30.0), (iii) light adapted 3.0 cd•s•m^-2^ at 2 Hz (LA 3.0), and (iv) light adapted 3.0 cd•s•m^-2^ 30 Hz flicker (LA 3.0 30 Hz). The extended protocol also included the recording of dark adapted responses to an intensity series of flashes in order to detect an excessive enlargement of dark adapted b-wave (patients 1 and 4).

The dark adapted b-wave amplitude elicited by a stimulus intensity 0.01 (DA0.01) was delayed and decreased in patients 3 and 4, but was normal but delayed in patient 1. The responses for DA0.01 were undetectable in patient 2. An excessive increase in the b-wave for the extended protocol was found in two patients, 1 and 4. In addition, the a-wave was square-shaped with a supernormal b-wave elicited by stimulus intensity 30.0 (DA 30.0) in all four patients. The photopic ERGs (LA 3.0 and LA 3.0 30Hz) were decreased in all four patients (Table 3 and Figure 2).

### Molecular genetics

The molecular genetic findings are summarized in Table 2 and Appendix 1. Likely disease-causing variants in *KCNV2* were identified in all four patients. The four likely disease-causing variants were p.Arg27His, p.Cys177Arg, p.Arg206Pro, and p.Gly461Arg (Appendix 1), and two likely non-disease-causing variants (polymorphisms) were p.Gly61Gly and p.Ala265Ala (Appendix 2). The segregation of each allele was confirmed by screening of other family members for all these variants.

**Figure 3:**
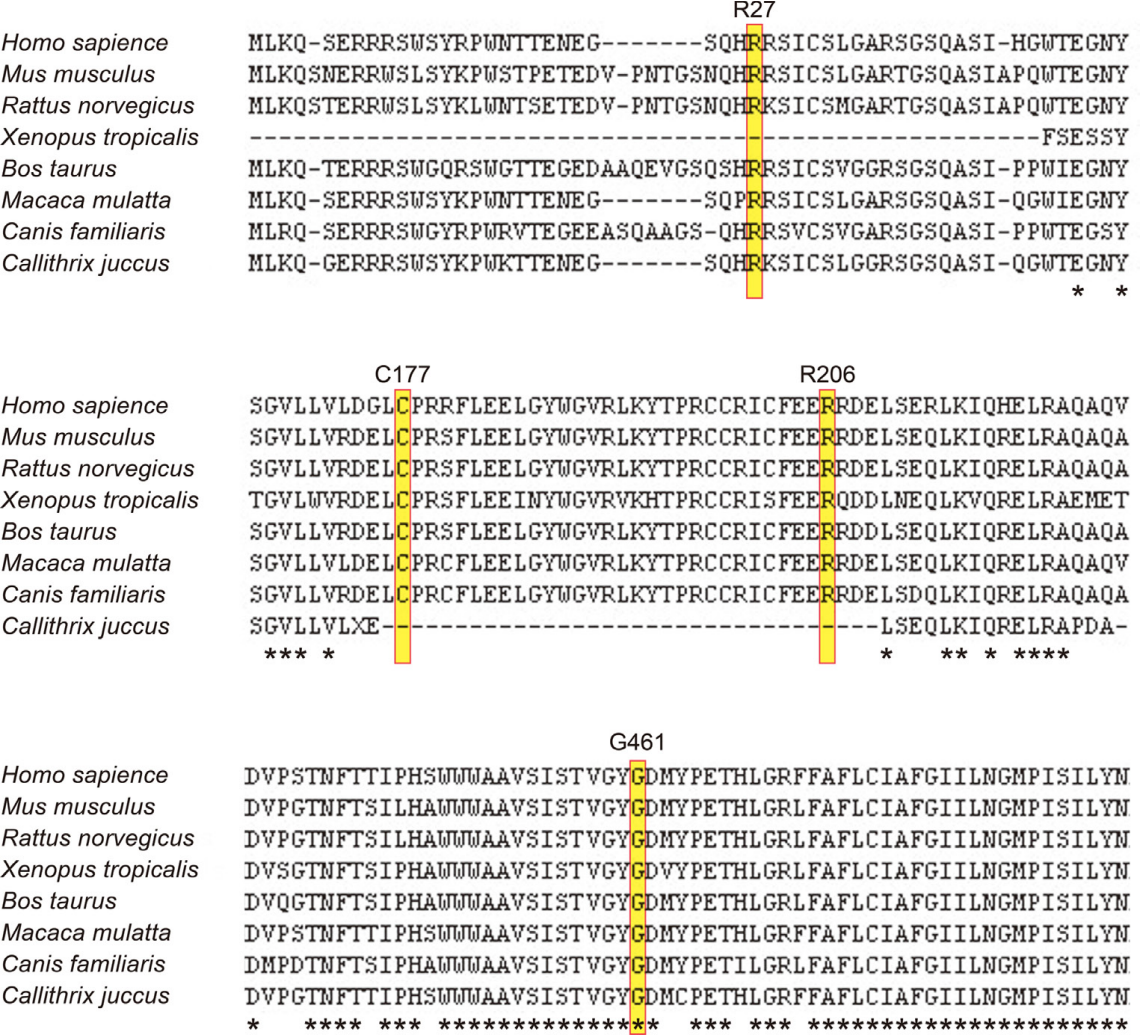
Multiple alignment of eight species of potassium channel, subfamily V, member 2 orthologs. The amino acid-sequence alignment is numbered in accordance with the *Homo sapiens* potassium channel, subfamily V, member 2 (*KCNV2*) sequence (ENSP00000371514). The positions of mutated residues, Arg27 (c.80 G>A, p.Arg27His), Arg177 (c.529 T>C, p.Cys177Arg), Arg206 (c.617 G>C, p.Arg206Pro), and Gly461 (c.1381 G>A, p.Gly461Arg), are highlighted. The alignment was performed with the Clustal Omega program, and the asterisk indicates a completely conserved residue.

Detailed molecular results including in silico analysis to assist in predicting the pathogenicity of the four disease-causing variants identified are shown in Appendix 1. All of the four likely disease-causing variants were single nucleotide changes with one amino acid substitution (missense), i.e., p.Arg27His, p.Cys177Arg, p.Arg206Pro, and p.Gly461Arg. Compound heterozygosity for the two alleles, p.Cys177Arg and p.Gly461Arg, in patients 1, 2, and 3 and homozygosity for the complex alleles, p.Arg27His and p.Arg206Pro, in patient 4 were revealed by the segregation analyses. The p.Gly461Arg variant has been reported, and the p.Arg27His, p.Cys177Arg, and p.Arg206Pro variants are putative novel. In silico analysis revealed an “intolerant” protein function or a “probably or possibly damaged” protein but no effect on splicing in the three putative novel variants (SIFT, Poplyphen2, and Human Splicing finder; Appendix 1). The reported missense variant, p.Gly461Arg, with possibly affecting splicing was detected in six out of 13,006 individuals of the Exome Variant Server; the three novel variants, p.Arg27His, p.Cys177Arg, and p.Arg206Pro, were not identified. Three missense variants, p.Arg27His, p.Cys177Arg, and p.Arg206Pro, were highly conserved among the orthologs, and one missense variant, p.Gly461Arg, was completely conserved (Figure 3).

**Figure 4:**
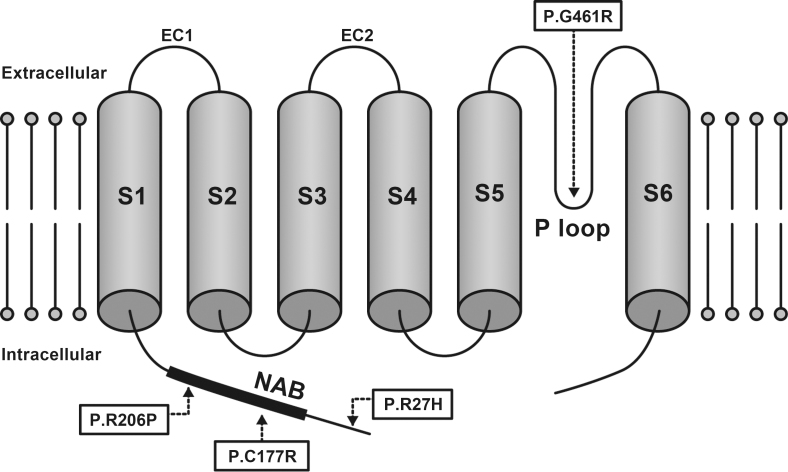
Model of the potassium channel, subfamily V, member 2 protein structure. A schematic representation of the potassium channel, subfamily V, member 2 (*KCNV2*) subunit of the K channel is drawn showing the approximate position of missense disease-causing variants identified in this study. The *KCNV2* protein consists of an N-terminus, an N-terminal A and B box (NAB), and six transmembrane domains (S1–S6), with two extracellular loops (EC 1, 2) and a K selective motif, GlyTyrGly, in the pore-forming loop (P loop) between S5 and S6.

A model of the *KCNV2* protein structure showing the approximate position of the missense disease-causing variants identified is presented in Figure 4. The KCNV2 protein comprises 545 amino acids and contains an N-terminal A and B box (NAB) and six transmembrane domains, (S1–S6), with a K selective motif, GlyTyrGly, in the pore-forming loop (P loop) between S5 and S6 [18]. One variant is located within the N-terminus (p.Arg27His), two variants, p.Cys177Arg and p.Arg206Pro, within the NAB, and one variant, p.Gly461Arg, within the P-loop.

Detailed molecular results of two non-disease-causing variants (polymorphisms) including the in silico analyses are summarized in Appendix 2. These two homozygous variants, p.Gly61Gly and p.Ala265Ala, were synonymous changes in the cording region and were predicted to be benign or have no effect on splicing (Polyphen2 and Human Splicing finder program analysis). Both were present in a high number of chromosomes in the Exome Variant Server database (7647/13006 for p.Gly61Gly and 5636/13006 for p.Ala265Ala, respectively).

## Discussion

Our results showed the molecular genetic characteristics of four Japanese patients with CDSRR, which, to the best of our knowledge, is the first report of these characteristics of *KCNV2* retinopathy in an East Asian population. Our four patients harbored the likely disease-causing variants in *KCNV2*. Compound heterozygosity for two alleles, p.Cys177Arg and p.Gly461Arg, in three patients and homozygosity for two complex alleles, p.Arg27His and p.Arg206Pro, in one subject were confirmed. Three of the four variants, p.Arg27His, p.Cys177Arg, and p.Arg206Pro, were novel, which indicates all genotypes identified in our series have never been described before.

The clinical and electrophysiological characteristics of our four patients were similar to those of reported patients [8-11,13,14,17,18]. Additionally, all four patients presented with a decrease in central vision whose onset was in the first decade of life with minimal fundus changes and a characteristic ring enhancement of the AF signal (Table 2 and Figure 1). These findings are also in accordance with earlier reports [9-12,14]. SD-OCT demonstrated a discontinuous or absent inner and outer segment junction line in two patients as previously reported [10]. In addition, the absence of the cone outer segment tip line at the macular region was also confirmed in all four patients.

The pathognomonic electrophysiological features were demonstrated in all four patients, viz., delayed and reduced photopic ERGs, delayed ERGs for DA 0.01, and a square-shaped a-wave with a supernormal b-wave for DA 30.0 (Table 3 and Figure 2). An excessive increase in the b-wave for the DA ERGs to an intensity series of flashes was also confirmed in patients 2 and 3. Therefore, the unique rod system abnormalities were identical to those reported for *KCNV2* retinopathy [9,14].

Compound heterozygosity for two alleles, p.Cys177Arg and p.Gly461Arg, was found in patients 1, 2, and 3. The p.Gly461Arg with relatively higher allele frequency affects the third residue of the ultraconserved-GYG-tripeptide motif that acts as an ion selectivity filter in the K channel’s pore-forming loop, P loop, between S5 and S6 (Figure 4) [30]. The clinical effect of p.Gly461Arg was well characterized earlier [10,16,17]. Friedburg et al. reported that three siblings with homozygous p.Gly461Arg had a relatively severe phenotype with an early onset and nystagmus at <5 years of age, visual acuity decrease (0.1–0.25, constantly), minimal fundus changes, ring enhancement at the foveal AF image, and an excessive increase in the b-wave for scotopic ERGs to an intensity series [17]. In contrast to the previous reports on homozygous patients, the three patients with heterozygous p.Gly461Arg in our series did not have nystagmus, and two of our patients had less severe BCVA decrease (0.7–0.8). These findings imply that the phenotype of the compound heterozygous for p.Gly461Arg and p Cys177Arg could have a less severe phenotype than those homozygous for p.Gly461Arg. It is of interest that the phenotypic spectrum, compound heterozygous for p.Gly461Arg and p Cys177Arg, was also observed in our series. Two relatively mild phenotypes were observed in the two siblings in our series (patients 1 and 2). In addition, one relatively severe phenotype, with more severe visual acuity decrease (0.1) and photoreceptor/RPE abnormalities at the macula, was detected in patient 3.

Three of the new disease-causing missense variants were located within the N-terminal region of the protein (Figure 4): p.Arg27His within the N-terminus and p.Cys177Arg and p.Arg206Pro within NAB. p.Cys177Arg was completely segregated, and the predicted pathogenesis and evolutionary conservation were confirmed. The coexistence of two likely disease-causing variants, p.Arg27His and p.Arg206Pro, on the same chromosome was also identified in our series with segregation analyses. The patient who was homozygous for these two complex variants had a severe phenotype, with an early onset (2 years), nystagmus, and severe visual acuity decrease (0.1 to 0.08). Both variants were predicted to be pathogenic with evolutionary high conservation (Appendix 1and Figure 3). Whether one of these variants is a neutral polymorphisms in cis with disease-causing one, or whether family 4’s alleles are complex with two independently damaging missense variants remains to be determined.

To conclude, this study further delineates the molecular genetic findings of *KCNV2* retinopathy. Three putative novel variants were identified in our four Japanese patients with CDSRR, and our findings suggest there may be a distinct spectrum of *KCNV2* alleles in the Japanese population. However, the clinical findings were similar to that of the reported other population. Electrophysiology was fundamental to the diagnosis with pathognomonic findings due to channelopathy. The pathognomonic characteristics may be a useful method of determining the success of clinical therapeutic trials with gene replacement or pharmacological treatments for channelopathy.

## Appendix 1. Results of in silico molecular genetic analysis of *KCNV2* mutations identified.

To access the data, click or select the words “Appendix 1.” Pt = patient; Hom = homozygous; Het = heterozygous; SIFT = sorting Intolerant from Tolerance; HSF = human splicing finder program; CV = consensus values; EVS = exome variant server; POD = possibly damaging; PRD = probably damaging; ND = not detected. SIFT (version 4.0.4) results are reported to be tolerant if tolerance index ≥ 0.05 or intolerant if tolerance index < 0.05. Polyphen-2 (vision 2.1) appraises mutations qualitatively as Benign, Possibly Damaging or Probably Damaging based on the model's false positive rate. The cDNA is numbered according to Ensemble transcript ID ENST00000382082, in which +1 is the A of the translation start codon. Human splicing finder version 2.4.1 was applied to predict the effect of each variant on splicing. The results from HSF matrix indicate the values for the wild type and mutant sequences. The larger difference of values between the wild type and the mutant sequences indicates the greater change that the variant can affect on the splice site. EVS denotes variants in the Exome Variant Server, NHLBI Exome Sequencing Project, Seattle, WA.

## Appendix 2. Molecular analysis of *KCNV2* polymorphisms.

To access the data, click or select the words “Appendix 2.” Pt = patient; Hom = homozygous; Het = heterozygous; SIFT = sorting Intolerant from Tolerance; HSF = human splicing finder program; CV = consensus values; EVS = exome variant server.
